# Association between Blood Omega-3 Index and Cognition in Typically Developing Dutch Adolescents

**DOI:** 10.3390/nu8010013

**Published:** 2016-01-02

**Authors:** Inge S. M. van der Wurff, Clemens von Schacky, Kjetil Berge, Maurice P. Zeegers, Paul A. Kirschner, Renate H. M. de Groot

**Affiliations:** 1 Welten Institute, Research Centre for Learning, Teaching, and Technology, Open University of the Netherlands, Heerlen 6419 AT, The Netherlands; Paul.Kirschner@ou.nl (P.A.K.); renate.degroot@ou.nl (R.H.M.G.); 2 Omegametrix, Martinsried 82 152, Germany; Clemens.vonSchacky@med.uni-muenchen.de; 3 Aker BioMarine Antarctic AS, Lysaker NO-1327, Norway; kjetil.berge@akerbiomarine.com; 4 NUTRIM School of Nutrition and Translational Research in Metabolism, Maastricht University, Maastricht 6200 MD, The Netherlands; Zeegerm.zeegers@maastrichtuniversity.nl; 5 CAPHRI School for Public Health and Primary Care, Maastricht University, Maastricht 6200 MD, The Netherlands

**Keywords:** docosahexaneoic acid (DHA), eicosapentaenoic acid (EPA), adolescents, cognition, Omega-3 fatty acids, Omega-3 Index

## Abstract

The impact of omega-3 long-chain polyunsaturated fatty acids (LCPUFAs) on cognition is heavily debated. In the current study, the possible association between omega-3 LCPUFAs in blood and cognitive performance of 266 typically developing adolescents aged 13–15 years is investigated. Baseline data from Food2Learn, a double-blind and randomized placebo controlled krill oil supplementation trial in typically developing adolescents, were used for the current study. The Omega-3 Index was determined with blood from a finger prick. At baseline, participants finished a neuropsychological test battery consisting of the Letter Digit Substitution Test (LDST), D2 test of attention, Digit Span Forward and Backward, Concept Shifting Test and Stroop test. Data were analyzed with multiple regression analyses with correction for covariates. The average Omega-3 Index was 3.83% (SD 0.60). Regression analyses between the Omega-3 Index and the outcome parameters revealed significant associations with scores on two of the nine parameters. The association between the Omega-3 Index and both scores on the LDST (β = 0.136 and *p* = 0.039), and the number of errors of omission on the D2 (β = −0.053 and *p* = 0.007). This is a possible indication for a higher information processing speed and less impulsivity in those with a higher Omega-3 Index.

## 1. Introduction

In recent decades, an increasing interest in the health benefits of long-chain polyunsaturated fatty acids (LCPUFAs) has been developed. Aside from its influence on cardiovascular health, it has also attracted attention because of its association with mental health (ADHD, autism, dyslexia) [1], cognitive functioning of healthy individuals [2,3,4] and cognitive decline in the elderly [5,6,7]. LCPUFAs and especially docosahexaneoic acid, 22:6*n*-3 (DHA), and eicosapentaenoic acid 20:5*n*-3 (EPA) are involved in many aspects of brain functioning such as neuronal membrane fluidity, neurotransmission, signal transduction, brain blood flow, and blood-brain barrier integrity [8,9]. The interest in the possible positive influence of LCPUFAs on brain functioning has led to a large number of both observational and experimental studies (for a review see [10,11]). These studies have, however, mainly focused on either diseased populations of infants, children, adults, and the elderly. Studies in typically developing adolescents are limited. The current study addresses this deficit.

Adolescence is a period in which LCPUFAs could be of special importance. During adolescence, the brain, especially the prefrontal cortex, undergoes development which continues until after age 20 [12,13]. The development of the prefrontal cortex is of utmost importance, since this development lays the basis for higher order cognitive functions that have been associated with academic achievements [14]. Moreover, the prefrontal cortex is a brain region especially enriched in DHA [15], and higher DHA intake has been associated with changes in the functional activity of the prefrontal cortex in boys aged 8–10 [16].

To our knowledge, three observational studies looking at the association between fish intake (the most important source of omega-3 LCPUFAs) and cognitive functioning in adolescents have been executed. Kim and colleagues showed that adolescents aged 15 years who regularly consumed fish had significantly better academic performance than peers who never or hardly ever consumed fish [17]. Aberg *et al.* demonstrated that high fish consumption in boys at age 15 was associated with better cognitive performance at age 18 [18]. Lastly, de Groot *et al.* studied 700 Dutch high school students aged 12–18 years. Fish consumption data, end term grades in Dutch, English and Math, scores on the Amsterdam Vocabulary Test, and scores on the Youth Self-Report (a self-reported measure for attention problems) were collected [19]. Results revealed that 13.6% of the Dutch adolescents never ate fish, 63.1% ate fish but too little to meet at least half of the recommended amount, 16.9% reached half of the recommended amount, and 6.4% met national guidelines (fish twice per week). Analysis of the variance showed significant differences between the four fish consumption groups (never, <1 per week (e.g., 1 time per month), 1 to 2 times per week, ≥2 times per week) in vocabulary, and a trend for significance was found for the average end term grade. Significant quadratic associations (u-shape association) between fish consumption, vocabulary (*p* = 0.01), and average end term grades (*p* = 0.001) were shown. Higher fish intake was associated with a more advanced vocabulary and an almost significantly higher average end term grade. However, eating more fish than the recommended amount (>2 fish portions/week) seemed to no longer be beneficial. Overall, the observational studies in adolescents point to a beneficial association between fish intake (the main source of the omega-3 LCPUFAs DHA and EPA) and school grades.

Fish consumption is the most important dietary source of LCPUFAs but not the only source [20]. Moreover, there is a large interpersonal variability in the uptake of LCPUFAs [21]. Thus, to be sure about the association between LCPUFAs and cognition in adolescents, measurement of LCPUFAs in blood is needed. Therefore, the main objective of this study is to investigate the association between the Omega-3 Index (EPA + DHA in erythrocytes as percentage of total fatty acids measured [22]) measured in blood and cognitive performance in typically developing adolescents of lower general secondary education (LGSE). Cognition is a very broad term that includes both lower order simple responses and higher order processes. The higher order processes are also called the executive functions, and it is generally agreed that there are three core executive functions namely: (i) inhibition and interference; (ii) working memory; and (iii) cognitive flexibility [23]. These executive functions are used to build higher order skills such as reasoning and problem solving. Therefore, the executive functions are important for academic success and cognitive development [23]. These executive functions are located in the prefrontal cortex, the brain area most in development during adolescence [24]. The cognitive tasks used in the current study are standard tasks of cognitive/executive functioning for this age group and have previously been shown to increase activation of the frontal cortex, the area of the brain associated with the accumulation of DHA [16].

In addition to the main objective, two sub-objectives will be addressed. A number of earlier studies have shown differences in the LCPUFA status between typically developing participants and participants with disorders such as ADHD, autism, and dyslexia [25,26]. However, to our knowledge, whether LCPUFAs are associated with cognition in participants with learning disorders differently than in those without learning disorders has not yet been assessed. The second objective of the current study is, therefore, to explore whether the association between the Omega-3 Index and cognitive ability is different between adolescents with and without learning disorders.

Social economic status, often operationalized as educational level, has been shown to be associated with diet quality (*i.e.*, people with a higher SES have better diet quality) [27]. Moreover, in adults, higher social economic status has been found to be associated with higher fish consumption [28]. However, even though it is known that students from lower general secondary education (LSGE) levels have a less healthy diet and lifestyle than students from the higher levels [29], how much fish students from the LSGE consume has, to our knowledge, not yet been assessed. Therefore, the third objective of this study is to explore the fish consumption of second year students of the LSGE.

## 2. Materials and Methods

### 2.1. Design

This study was part of a larger randomized controlled clinical trial (Food2Learn) studying the influence of omega-3 LCPUFA supplementation on cognitive performance, mental wellbeing, and academic achievement scores in adolescents attending LGSE. Baseline data of Food2Learn were used to study the association between the Omega-3 Index measured in whole blood and cognition. Food2Learn has been approved by the Medical Ethical Committee of Atrium-Orbis-Zuyd Hospital (now Zuyderland), Heerlen, The Netherlands (NL45803.096.13). Food2Learn has been registered at the Netherlands Trial Register (NTR4082), which is connected to Clinicaltrials.gov (registered as NCT02240264.)

### 2.2. Procedure and Participants

Participants were recruited from 17 schools in the south of the Netherlands. For students who wanted to participate, an informed consent form had to be signed by themselves as well as by both parents and/or guardians. After informed consent was received, students underwent a finger prick to measure their Omega-3 Index. Inclusion criteria for Food2Learn were: 1 Omega-3 Index <5%, as it was expected that omega-3 fatty acid supplementation will be especially beneficial for participants with a very low baseline Omega-3 Index [22]; and 2 attending the second year of LSGE because Richardson *et al.* showed that omega-3 supplementation was especially beneficial in the 20% lowest performing students [30]. Therefore, the choice for students at one of the lowest educational levels in The Netherland’s LGSE was made. In the Netherlands, secondary education is divided into three levels: pre-university, higher general secondary education, and LGSE. Approximately 38% of all adolescents follow LGSE [31]. LGSE is further divided up into four sublevels. For this study, students from the highest sublevel, the theoretical learning pathway (TLP), were recruited. Approximately 40% of students attending LGSE are in the TLP [31]. No other inclusion criteria were applied, thus, all second year students of the LSGE with an Omega-3 Index <5% could participate. After inclusion, participants underwent a neuropsychological test battery in a small group setting (10 students max) consisting of: Letter Digit Substitution Task (LDST), D2 test of Attention (D2), Digit Span Forward (DSF), and Backward (DSB). In addition, they filled out a number of questionnaires to collect important background information. The tests were led by one researcher via a standardized protocol, while one or two other researchers (depending on the group size) were monitoring to ensure that participants understood the tests and complied with the protocol. Before continuing with the real tests, students received a practice version of the tests, feedback was given, and the students confirmed they understood the tests. After this group test session, all participants filled out a questionnaire individually (data not used in the current study), during which participants were called one by one to perform the individual neuropsychological tests: Stroop Test and Concept Shifting Test (CST) under the supervision of one researcher.

### 2.3. Dependent Variable—Blood Analysis

Whole blood was obtained from a finger prick with an automated lancet and directly transferred to a filter paper (Whatman 903, General Electric, Frankfurt, Germany) pre-treated with a stabilizer. Filter papers were shipped immediately to Omegametrix, Martinsried, Germany for analysis. Whole blood fatty acid compositions were analyzed according to the HS-Omega-3 Index methodology [32]. Fatty acid methyl esters are generated by acid transesterification and analyzed by gas chromatography using a GC2010 Gas Chromatograph (Shimadzu, Duisburg, Germany) equipped with a SP2560, 100-m column (Supelco, Bellefonte, PA, USA) using hydrogen as a carrier gas. Fatty acids are identified by comparison with a standard mixture of fatty acids. Results are given as EPA plus DHA expressed as a percentage of total identified fatty acids after response factor correction. Since the Omega-3 Index is defined as EPA + DHA in erythrocytes, it was calculated using a sliding correction factor. The coefficient of variation for EPA plus DHA typically is 5%. Analyses are quality-controlled according to DIN ISO 15189.

#### 2.3.1. Independent Variables—Cognitive Measures

The LDST is a paper-pencil task used to measure speed of information processing [33]. A nine letter/digit key is noted at the top of a page. Below this key, rows of letters are printed, and participants are asked to write the corresponding number in the box underneath the letter as quickly as possible. The number of correctly filled in numbers in 60 s is used as a measure of speed of information processing.

#### 2.3.2. D2 Test of Attention

The D2 test is a paper-pencil task used to measure selective attention [34]. Participants are presented with 14 rows each consisting of 47 stimuli. Stimuli are the letters d and p with a varying number of dashes (between 1 and 4), below, above, or on both sides. Participants are instructed to only cross out the d with two dashes (2 above, 2 below or one on both sides) and ignore all other stimuli. Participants have to process as many stimuli as possible in 20 s per line after which they have to continue with the next row without pausing. The following measures per row and in total are noted after completion: total number of stimuli processed, number of correctly crossed out d2’s, number of d2’s not crossed out, and number of stimuli wrongly crossed out (thus, non d2). The total number of stimuli processed is used as a measure for information processing speed. The number of target stimuli not crossed out (*i.e.*, errors of omission) and non-target stimuli crossed out (*i.e.*, errors of commission) are used as a measure for impulsivity and inattention, respectively.

#### 2.3.3. Digit Span Forward and Backward

The DSF is a measure for short-term memory that primarily activates the phonological loop. The DSB activates the executive component directly and shows the dynamic relationship between passive storage and active manipulation or transformation of information held in the memory [35] and is thus a measure for working memory (the ability to hold information in the mind and work with it). The DSF consists of 12 sequences of digits varying in length from three to eight digits (each length twice). Digits are announced by the researcher at a rate of approximately one digit per second. After completion of the digit sequence, participants are asked to write down the sequence. The DSB is similar to the DSF, except for the fact that it consists of 12 digit sequences varying in length from two to seven digits (each length twice) and after completion of the sequence by the researcher, students are asked to write down the sequence backwards, starting with the last number announced. The longest sequence of numbers of which participants had correctly written down at least one of the two rows was used as a measure for working memory.

#### 2.3.4. Concept Shifting Task

The CST is a measure for cognitive shifting [36]. Cognitive shifting is the ability to adapt to changes in the environment by switching from one mental set to another [33]. The task consists of four parts. All parts consist of a sheet of paper with 16 small circles grouped in one large circle. In task A, the small circles are randomly filled with numbers, in task B the circles are filled with letters, and in task C the circles are filled with both. Participants are asked to cross out the items in the correct order (A: 1 to 12; B: A to P; C: 1–A–2–B to 8-H). Lastly, there is Task Zero, which consists of empty circles, where participants are asked to cross out the circles as quickly as possible. Task Zero is administered twice, and the average of these times is used to correct for basic motor speed in the other tasks. For all tasks, the time taken to complete and the number of errors are noted. The average of motor-speed corrected time needed for A and B was subtracted from the motor speed corrected time needed for C and used as a measure for shifting.

#### 2.3.5. Stroop Test

The Stroop test provides a measure for cognitive inhibition. Cognitive inhibition is the ability to inhibit an overlearned response in favor of a more unusual one [37]. The Stroop task, as used in Food2Learn, consists of three cards containing 40 stimuli each: color names printed in black (Task 1), colored patches (Task 2), and color names printed in congruent or incongruent color (Task 3). For Task 1, participants are asked to read the name out loud. For Task 2, participants name the color of the patches, and for Task 3, participants name the ink color the word is printed in. Task 3 is a measure of mental flexibility and the ability to inhibit a dominant response (reading). The time needed for Task 2 was subtracted from the time needed for Task 1, the result of this sum was subtracted from the time needed for Task 3. The result of this sum was used as a measure for inhibition.

#### 2.3.6. Additional Measures

Students filled out a questionnaire to assess covariates. The following covariates were assessed as they are known to correlate with cognition: BMI (weight/length^2^, self-reported) [36], sex [37], age [38], alcohol consumption [39], smoking [40], and parental level of education [41]. Alcohol consumption was assessed with two questions: the number of days/week the participant generally drank alcohol and the number of units the participant drinks on a day that (s)he drinks alcohol. Alcohol consumption was defined as the number of alcohol units/time multiplied by the number of drinking days/week, and the measurement was used as a continuous measure. Smoking was assessed with the question: “How many cigarettes do you smoke per week?’’. If the participant indicated consuming cigarettes, (s)he was classified as smoker. Parental level of education was filled out by the parents on an ordinal eight-point scale [42]. Parental level of education was defined as the parent with the highest level of education, which is an indication for social economic status [43]. Additionally, fish consumption was assessed with a short, validated, and self-reported questionnaire [3]. Different kinds of fish were divided based on their DHA content: low (fish fingers, prawns, pickled herring, cod, mussels, plaice, tuna, tilapia); medium (trout, raw herring, smoked eel, smoked salmon, canned salmon); and high (smoked herring, herring and tomato sauce, mackerel, canned sardines, salmon). The consumption (never, once a month, two to three times a month, once a week or more than once a week) was used to calculate the fish consumption score. For the low DHA fish 0, 1, 2, 4, 8 points; for the medium DHA fish, 0, 2, 4, 8, 16 points; and for the high DHA fish, 0, 3, 6, 12, 24 points. The score for fish consumption could thus vary between 0 and 48 points. Lastly students were asked to indicate whether they had a disorder which could influence learning (examples were given) and who had made that diagnosis.

### 2.4. Quality Control

To ensure the quality of the data, all tests were scored by two independent researchers. Any discrepancies were solved by discussion. Furthermore, in order to prevent typing mistakes, all data were entered in the database twice, after which the two files were automatically compared. Any discrepancies between the two data files were checked and corrected by a third researcher.

### 2.5. Statistical Analyses

Data were checked for normality and if necessary, transformation was applied. Data were analyzed with linear regression or generalized linear regression (Poisson) for count data and data with a skewed distribution. For all analyses, first, a model with all covariates (*i.e.*, smoking, alcohol consumption units per week), BMI, age, level of parental education, sex, and diagnosis) was built; Model A. In Model B, the Omega-3 Index was added. In a separate analysis, potential moderation between the Omega-3 Index and diagnosis was tested. If results were significant, a sub-group analysis for typically developing adolescents and those who had indicated to have some sort of learning disorder (autism, dyslexia, ADHD, *etc.*) were executed in the same way (diagnosis was not entered as a covariate). For all analyses, a *p*-value below 0.05 was considered to be significant. All analyses were carried out using SPSS statistics version 22.

## 3. Results

### 3.1. Participants

A total of 286 students consented to participate in the study. Of these, four dropped out before blood sampling due to personal reasons and 16 had an Omega-3 Index > 5%. Thus, the associations between the Omega-3 Index and cognition of 266 participants (127 boys, 139 girls; *M_age_* = 14.1 years) are discussed in this paper. Characteristics of the participants can be found in Table 1. Omega-3 Index and LCPUFAs as determined in blood can be found in Table 2. Scores on the cognitive tests can be found in Table 3. In this sample, 69 participants indicated having a disorder which can impact learning; 14 indicated having Attention Deficit Hyperactivity Disorder (ADHD) or Attention Deficit Disorder (ADD); 45 indicated having dyslexia or dyscalculia; eight reported an autism spectrum disorder; and two indicated a depression. In the total sample of 266 adolescents, 13.8% indicated never consuming fish, 77% indicated eating fish very irregularly (*i.e.*, less than half of the recommend amount of 450 mg DHA + EPA per day), 8.4% consumed at least half of the recommended amount (once a week), and 1% indicated consuming fish more than once a week. There was a significant difference in fish consumption between boys and girls (*p* = 0.024), with boys consuming more fish than girls. However, this did not result in significant differences in the Omega-3 Index (*p* = 0.561). The total score on the fish questionnaire correlated significantly with both the Omega-3 Index (*n* = 216, *r* = 0.294, *p* < 0.001) and DHA concentration (*n* = 216, *r* = 0.287, *p* < 0.001).

### 3.2. Cognitive Performance

Analyses revealed a significant association between the Omega-3 Index and score on the LDST (β = 0.136, *p* = 0.039). The addition of the Omega-3 Index to the model increased the *r*^2^ with 0.017 (Table 4), *i.e.*, an additional 1.7% of the variance was explained. Furthermore, a significant association between the Omega-3 Index and errors of omission on the D2 was shown (β = −0.053, *p* = 0.007) (Table 5). The analysis for errors of omission also showed a significant moderator effect (*p* = 0.005). No other significant associations between the Omega-3 Index and any of the other cognitive measures were found.

**Table 1:** Participant characteristics.

Characteristic	All Participants		With Diagnosis ^1^		Without Diagnosis ^2^		*p*-Value ^5^
	Mean ± SD or *N* (%)	*N*	Mean ± SD or *N* (%)	*N*	Mean ± SD or *N* (%)	*N*	
Age (years)	14.10 ± 0.49	266	14.26 ± 0.51	69	14.05 ± 0.47	196	**0.002**
Male/Female	127/139 (47.7/52.3%)	266	36/33 (52.2/47.8%)	69	93/103 (47.5/52.5%)	196	0.499
Smoking no/yes ^3^	239/26 (90.2/9.8%)	265	59/10 (85.5/14.5%)	69	179/16 (91.8/8.2%)	195	0.132
Body Mass Index (BMI)	19.92 ± 3.00	248	20.34 ± 3.61	65	19.77 ± 2.74	183	0.187
Alcohol units per week ^4^	0.46 ± 1.77	266	0.69 ± 2.85	69	0.39 ± 1.19	196	0.218
Level of Parental Education (LPE)	5.07 ± 1.52	248	5.21 ± 1.40	66	5.02 ± 1.56	182	0.371

^1^ Diagnosis was defined as a diagnosis possible to influence learning; this was indicated by students themselves and included (but not limited to) dyslexia, dyscalculia, depression, autism, and Attention Deficit Hyperactivity Disorder (ADHD); ^2^ without diagnosis was defined as all students who did not indicate to have a diagnosis; ^3^ smoking was defined as anybody who indicated to smoke more than 0 cigarettes per week; ^4^ alcohol units per week was operationalized as number of day per week that alcohol is consumed times units per consumption moment; ^5^ comparison between those with and those without diagnoses. ANOVA was used for age, BMI, LPE and alcohol units per week, Chi Square for smoking, and sex. Significant differences (*p* < 0.05) are noted in bold.

**Table 2:** Fatty acid blood.

Fatty Acid (% wt/wt of Total FA)	All Participants	With Diagnosis ^1^	Without Diagnosis ^2^	*p*-Value ^3^
	*N* = 261	*N* = 68	*N* = 193	
	Mean ± SD	Mean ± SD	Mean ± SD	
Omega-3 Index	3.83 ± 0.60	3.79 ± 0.61	3.84 ± 0.60	0.537
DHA 22:6*n*-3	2.58 ± 0.49	2.56 ± 0.50	2.59 ± 0.49	0.667
EPA 20:5*n*-3	0.39 ± 0.16	0.38 ± 0.13	0.39 ± 0.16	0.356
AA 20:4*n*-6	11.19 ± 1.25	11.49 ± 1.34	11.08 ± 1.20	**0.022**
ObA 22:5n-3	0.43 ± 0.10	0.43 ± 0.11	0.44 ± 0.10	0.725

^1^ Diagnosis was defined as a diagnosis possible to influence learning; this was indicated by students themselves and included (but not limited to) dyslexia, dyscalculia, depression, autism and ADHD; ^2^ without diagnosis was defined as all students who did not indicate to have a diagnosis; ^3^ Comparison between those with and those without diagnoses. Significant differences (*p* < 0.05) are noted in bold. DHA: docosahexaneoic acid; EPA: eicosapentaenoic acid.

**Table 3:** Scores on the cognitive tests.

Measures	All Participants	With Diagnosis ^1^	Without Diagnosis ^2^	*p*-Value ^3^
	*N* = 261	*N* = 68	*N* = 196	
	Mean ± SD	Mean ± SD	Mean ± SD	
LDST (number)	34.47 ± 5.46	33.52 ± 6.51	34.80 ± 5.02	0.094
D2-correct (number)	163.13 ± 22.95	160.04 ± 24.24	164.22 ± 22.45	0.194
D-error of omission (number)	11.83 ± 10.73	11.25 ± 8.07	12.04 ± 11.53	0.598
D2-error of commission (number)	1.31 ± 10.73	1.54 ± 1.96	1.22 ± 1.43	0.161
D2-Total (number)	417.33 ± 56.46	408.93 ± 55.11	420.29 ± 56.77	0.151
Shifting score (s)	11.70 ± 6.83	11.69 ± 6.50	11.71 ± 6.96	0.980
Inhibition score (s)	31.35 ± 8.50	34.85 ± 9.19	30.12 ± 7.91	**0.000**
Digit span Forward (digits)	5.58 ± 0.88	5.26 ± 0.87	5.70 ± 0.85	0.616
Digit Span Backward (digits)	4.56 ± 0.98	4.51 ± 0.93	4.58 ± 1.00	**0.000**

^1^ Diagnosis was defined as a diagnosis possible to influence learning; this was indicated by students themselves and included (but not limited to) dyslexia, dyscalculia, depression, autism, and ADHD; ^2^ without diagnosis was defined as all students who did not indicate to have a diagnosis; ^3^ Comparison between those with and those without diagnoses. Significant differences (*p* < 0.05) are noted in bold.

**Table 4:** Results of multiple linear regression analyses between the Omega-3 Index and score on the Letter Digit Substitution Test (LDST) in the complete sample.

Predictor Variable	Β (Standardized) ^1^	Significance ^2^
*Model A (r^2^ = 0.058, df = 7, p = 0.051)*		
Smoking	0.028	0.679
Alcohol consumption	0.031	0.649
BMI	0.089	0.171
Age	0.047	0.477
Sex	0.177	**0.007**
Highest LPE	−0.056	0.387
Diagnosis	−0.104	0.113
*Model B (r^2^ = 0.075, df = 8, p = 0.019)*		
Smoking	0.031	0.643
Alcohol consumption	0.045	0.500
BMI	0.080	0.218
Age	0.036	0.584
Sex	0.172	**0.008**
Highest LPE ^3^	−0.084	0.203
Diagnosis	−0.094	0.147
Omega-3 Index	0.136	**0.039**

^1^ Standardized beta refers to how many standard deviations the dependent variable will change per standard deviation change in the predictor variable. Smoking, sex, and diagnosis were not standardized as they are dichotomous variables; ^2^ Significant results (*p* < 0.05) are printed in bold; ^3^ LPE = level of parental education.

**Table 5:** Results of generalized linear model analyses between the Omega-3 Index and number of errors of omission on the D2 test in the complete sample.

Predictor Variable	Β (Standardized) ^1^	Significance ^2^
*Model A (χ^2^ = 47.90, df = 7, p < 0.001)*		
Smoking	0.066	0.310
Alcohol consumption	0.036	**0.030**
BMI	0.043	**0.026**
Age	0.036	0.068
Sex	−0.047	0.226
Highest LPE	−0.087	**0.000**
Diagnosis	−0.071	0.109
*Model B (χ^2^ = 51.852, df = 8, p < 0.001)*		
Smoking	0.062	0.349
Alcohol consumption	0.030	0.078
BMI	0.043	**0.028**
Age	0.041	0.037
Sex	−0.052	0.181
Highest LPE ^3^	−0.077	**0.000**
Diagnosis	−0.083	0.063
Omega-3 Index	−0.053	**0.007**

^1^ Standardized beta refers to how many standard deviations the dependent variable will change per standard deviation change in the predictor variable. Smoking, sex, and diagnosis were not standardized as they are dichotomous variables; ^2^ Significant results (*p* < 0.05) are printed in bold; ^3^ LPE = level of parental education.

### 3.3. Sub-Group Analyses

When participants were divided into those without learning disorders and those who indicated having one or more learning disorders, differences between the two groups arose. Those with a diagnosis were significantly older (Table 1, *p* = 0.002, 14.26 ± 0.51, and 14.05 ± 0.47, respectively) than those without a diagnosis. Furthermore, they had a slightly higher AA status (Table 2, 11.08 ± 1.20, and 11.49 ± 1.34, respectively). With regard to the test scores, there was a significant difference in average score between those with and those without a diagnoses in inhibition as measured with the Stroop test (*p* = 0.000, 34.85 ± 9.19, and 30.12 ± 7.91, respectively) and on the digit span backwards (*p* = 0.000, 5.26 ± 0.869, and 5.7 ± 0.851, respectively).

When a moderation term was added to the regression analysis, a moderation effect was seen for the number of errors of omission (*p* = 0.005), *i.e.*, the association between the Omega-3 Index and score on the D2—the errors of omission were different between those with and those without diagnosis. Therefore, a separate group regression analysis was executed. This analysis showed no significant associations in adolescents with one or more learning disorders between the Omega-3 Index and errors of omission (*p* = 0.073). For typically developing adolescents, a significant association between the Omega-3 Index and errors of omission was seen (Table 6), students with a higher Omega-3 Index had a lower number of errors.

**Table 6:** Results of generalized linear model analyses between the Omega-3 Index and number of errors of omission on the D2 test in the typically developing participant sample.

Predictor Variable	Β (Standardized) ^1^	Significance ^2^
*Model A (χ^2^ = 42.11, df = 6, p < 0.001)*		
Smoking	0.032	0.685
Alcohol consumption	0.036	0.277
BMI	0.091	**0.000**
Age	0.002	0.914
Sex	−0.136	**0.003**
Highest LPE	−0.085	**0.000**
*Model B (χ^2^ = 55.642, df = 7, p < 0.001)*		
Smoking	0.029	0.714
Alcohol consumption	0.027	0.410
BMI	0.089	**0.000**
Age	0.015	0.515
Sex	−0.138	**0.002**
Highest LPE ^3^	−0.067	**0.003**
Omega-3 Index	−0.083	**0.000**

^1^ Standardized beta refers to how many standard deviations the dependent variable will change per standard deviation change in the predictor variable. Smoking and sex were not standardized as they are dichotomous variables; ^2^ Significant results (*p* < 0.05) are printed in bold; ^3^ LPE = level of parental education.

## 4. Discussion

The main aim of this study was to investigate the association between the Omega-3 Index measured in blood and cognitive performance of 14-year-old Dutch adolescents. The Omega-3 Index was significantly associated with information processing operationalized as LDST score. This indicates that a higher Omega-3 Index was associated with better information processing speeds. Every 1% increase in the Omega-3 Index was associated with an increase of 1.23 digits on the LDST. Also, students with a higher Omega-3 Index had fewer errors of omission on the D2 test of attention, an indicator of inattention/impulsivity (*i.e.*, they paid more attention than students with a lower Omega-3 Index). An increase of 1% in the Omega-3 Index was associated with a decrease of 0.94 stimuli forgotten to cross out. Associations with all other cognitive measures were not significant.

To our knowledge, this is the first study assessing the association between the Omega-3 Index measured in blood and cognition in typically developing adolescents from the general population. There are a number of observational studies of adolescents that found positive associations between fish consumption, the most important source of omega-3 LCPUFAs, and school grades [17,18,19]. However, even though cognition/executive functioning and school performance are correlated, they are not equal. School performance depends on additional factors such as time spent on homework [44] and personality [45]. Although we are not aware of studies looking at the association/relationship between LCPUFA status and cognition in adolescents, multiple studies of children are available. For example, Portillo-Reyes *et al.* found an improvement in processing speed in their supplementation study (180 mg DHA and 270 mg EPA per day for three months) of marginally malnourished children age 8–12 years [46]. Parletta *et al.* also found a positive effect of supplementation (750 mg EPA + DHA per school day for 40 weeks) on a non-verbal cognitive test [47]. However, there are also a number of studies that do not show an association or relationship between omega-3 LCPUFAs and cognition in children [48,49,50]. Overall, results remain mixed, and a number of possible explanations for these differences have been proposed [50,51]. For example, it has been suggested that an effect of LCPUFAs on cognition might be more likely to be demonstrated in underperforming children and adolescents, as shown in the study of Richardson *et al.* [30]. We tried to address this in the current study by recruiting students from one of the lowest educational levels in the Netherlands. Additionally, it has been suggested that LCPUFAs might only be beneficial in certain periods of life when the brain is developing, the so-called windows of opportunity. We tried to address this by including adolescents because the brain undergoes profound development in adolescence [12].

A number of earlier studies have shown a positive relationship between LCPUFAs and cognition in people with learning disorders [30,52,53,54]. Therefore, a moderator analysis was executed to check whether the association between the Omega-3 Index and score on the cognitive test was different between those with and those without a learning disorder. If a moderator effect was shown, separate analyses for adolescents who indicated to have a learning disorder *versus* typically developing adolescents were executed. There was a significant association between the covariate diagnosis and score on errors of commission and on the interference score. The moderator effect could, however, only be shown for errors of omission. Firstly, the number of students with a diagnosis was relatively low (*n* = 69), which could have led to a reduced statistical power. Secondly, the self-reporting of diagnosis and the fact that many adolescents did not know who made the diagnosis could have led to attenuation of the associations. Thus, the measure of diagnosis might not be accurate. However, when the test scores of those with and those without a diagnosis were compared, students with a diagnosis score lower on the test of interference (Stroop). This would suggest that the assessment of a learning disorder is accurate, since it has been shown before that patients with ADHD and other psychiatric problems have impaired performance on this test [55]. Moreover, the variation in the Omega-3 Index (inherent to our pre-selection of participants with an Omega-3 Index < 5%) was relatively low (SD = 0.61), which makes the appearance of associations less likely. In contrast, even though this spread was also low (SD = 0.60) in typically developing adolescents, a significant association between Omega-3 Index and cognitive measures could be shown. This could be explained by the fact that the number of students with a diagnosis was only 69; therefore, the power to detect an association was not sufficient [56].

The Omega-3 Index (3.83%) in this sample was relatively low (well below the recommended range of 8%–11% [22]). This could be due to the exclusion of participants with a high Omega-3 Index, although if these were included the mean was still only 3.89 (SD 0.67). The low Omega-3 Index in this sample is no surprise since 13.9% of the students did not consume any fish and 77% consumed fish rarely, as measured by the fish consumption questionnaire. This frequency of fish consumption is somewhat lower than the consumption of the adolescents in the sample of de Groot *et al.* [19]. However, the study of de Groot *et al.* was carried out with students in higher general secondary education or pre-university education with a somewhat higher social economic status (assessed by level of education of the parents) than the students in the current study. The number of students that never consume fish is also in line with the results from the National Dutch Consumption Survey, which indicates that 11% of boys and 18% of girls never consume fish [57]. Similarly in our sample, girls also consumed significantly less fish than boys. However, the number of adolescents who consumed fish twice or more a week was in only 1% in this sample, while in the survey 9% of the boys and 7% of the girls consumed the recommended amount of fish.

The main strength of the current study is that the Omega-3 Index was measured in blood. Furthermore, standardized and validated cognitive tests that assess several aspects of executive functioning were used. The main limitation of the study is that it is an observational study and can, therefore, not prove causality. Also, the variation in the Omega-3 Index was rather small. Furthermore, no Bonferroni correction for multiple statistical was applied, with correction significant results were not present anymore, which weakens the certainty of the associations found. However, the data presented here are part of a large intervention study, which will elucidate the effect of LCPUFA supplementation on cognition, mood, and academic achievement in adolescence. Furthermore, the supplementation study will achieve a higher Omega-3 Index and a larger spread in the Omega-3 Index, which could lead to more significant results (a number of associations were borderline significant).

In conclusion, this study has revealed a positive association between the Omega-3 Index measured in blood from typically developing adolescents and two of the nine cognitive measures. The results of the supplementation study will further elucidate the effect of LCPUFA supplementation on cognition. If a positive effect of LCPUFA supplementation on cognition is shown, this could help improve cognitive functioning and possibly the school performance of adolescents in a relatively inexpensive and easy way.
